# Data Augmentation of Backscatter X-ray Images for Deep Learning-Based Automatic Cargo Inspection

**DOI:** 10.3390/s21217294

**Published:** 2021-11-02

**Authors:** Hyunwoo Cho, Haesol Park, Ig-Jae Kim, Junghyun Cho

**Affiliations:** 1 Center for Artificial Intelligence, Korea Institute of Science and Technology, Seoul 02792, Korea; hwcho@kist.re.kr (H.C.); haesol@kist.re.kr (H.P.); drjay@kist.re.kr (I.-J.K.); 2 Division of Nano & Information Technology, KIST School, University of Science and Technology, Seoul 02792, Korea

**Keywords:** backscatter X-ray, data augmentation, cargo inspection, generative adversarial network, image translation

## Abstract

Custom inspection using X-ray imaging is a very promising application of modern pattern recognition technology. However, the lack of data or renewal of tariff items makes the application of such technology difficult. In this paper, we present a data augmentation technique based on a new image-to-image translation method to deal with these difficulties. Unlike the conventional methods that convert a semantic label image into a realistic image, the proposed method takes a texture map with a special modification as an additional input of a generative adversarial network to reproduce domain-specific characteristics, such as background clutter or sensor-specific noise patterns. The proposed method was validated by applying it to backscatter X-ray (BSX) vehicle data augmentation. The Fréchet inception distance (FID) of the result indicates the visual quality of the translated image was significantly improved from the baseline when the texture parameters were used. Additionally, in terms of data augmentation, the experimental results of classification, segmentation, and detection show that the use of the translated image data, along with the real data consistently, improved the performance of the trained models. Our findings show that detailed depiction of the texture in translated images is crucial for data augmentation. Considering the comparatively few studies that have examined custom inspections of container scale goods, such as cars, we believe that this study will facilitate research on the automation of container screening, and the security of aviation and ports.

## 1. Introduction

Globally, X-ray imaging has been utilized for customs inspection. This process allows for the detection of inner components of an object nondestructively; hence, it can be used for detecting smuggled, fraudulent, and dangerous goods, which are often hidden intentionally. The inspection process, however, requires considerable time and effort from human experts, and even experts find it difficult sometimes, depending on the equipment and cargo items. Owing to insufficient manpower and equipment, it was reported that a mere 1.6% of containerized cargo was inspected by using X-ray scanners in major South Korean ports [1].

To solve this problem, automated cargo inspection methods using artificial intelligence (AI) technology are being considered [2,3]. However, compared to other fields, it has not been actively researched [4] because it is difficult to obtain cargo X-ray image data. Compared to visible-light images, X-ray images are relatively limited, as very few people can own and operate X-ray equipment; in fact, most of the X-ray equipment that can scan cargo is owned by customs.

Researchers have widely used data augmentation to overcome the problem caused by insufficient training data. In previous research, for example, data augmentation was performed using classical image processing techniques for cargo inspection [5,6,7,8,9,10,11]. Objects to be detected, such as prohibited items, were cropped from the given X-ray image, the background was removed, and the extracted object was synthesized into an arbitrary container image to create a synthesized image [5,6,7,8,9,10]. This method can be useful for detecting objects in various backgrounds in the real environment. The limitation of such classical data augmentation lies in the diversity of objects. The network model learned from it may have difficulties in detecting various objects of different shapes and types in the actual test environment.

One possible approach to solving the aforementioned issue is by using image-to-image translation for data augmentation. The goal of image-to-image translation is to alter the characteristics or styles of an image while ensuring that the desired contents are unchanged. When it is applied to data augmentation for image classification or object detection, the contents related to the label information remain unchanged while other characteristics, such as domain-specific textures, colors, noises, or backgrounds, are adapted. This type of data augmentation is especially valid when the labeled source domain data and unlabeled target domain data are abundant, and labeled target domain data are relatively rare, which is the case for X-ray image processing in customs inspections. Therefore, this paper focuses on applying image-to-image translation to data augmentation and improving the method to make it suitable for backscatter X-ray (BSX) images acquired from ZBV (Z-backscatter van) [12]. It is noteworthy that it is almost impossible to acquire all the BSX data of the existing vehicle models, and worse, many new vehicle models are released every year from various manufacturers. Conversely, the RGB image of the vehicle can be easily obtained. If these RGB images are converted to BSX, we can use the data for network learning.

The direct application of a basic image-to-image translation method for generating BSX images, however, results in unsatisfactory results. Compared with real BSX images, fake images are blurred, losing the BSX-like noise patterns in both the foreground and background; meaningful details of the structures of the cars are also lost. We attribute this failure to the use of deterministic mapping in a largely underdetermined problem. Unlike the X-ray images used in medical image analysis, the BSX images for customs inspection have random background clutter, the structure and composition of objects are diverse, and different objects are randomly posed with possible overlaps. All these factors make the image translation from semantic mask images to X-ray images a one-to-many mapping process. As the vanilla pix2pix [13] creates a deterministic one-to-one mapping, it averages out the small details from overlapping objects or textures from X-ray noises into blurry or flat patches. Although the authors in [13] attempted to add random latent variables to make the mapping many-to-many or stochastic, the network was trained to ignore these additional variables.

In the context described above, this paper introduces a simple and effective image-to-image translation method that takes an additional texture image as an input to the image translation network. Unlike the random pixelwise noise variables, the actual texture images from the target domain can provide direct clues for the reconstruction of X-ray specific noise patterns, which correspond to the presence of actual matter or structured sensor noise. The close relationship between the texture input and output X-ray images helps the network learn to utilize it more easily during training. As a result, the generated X-ray images have better visual quality than those generated by the vanilla pix2pix [13]. In addition, we can generate diverse target images using a single image source by feeding the network with different texture images during the test phase. The synthesized images demonstrate different noise patterns and background clutter depending on the texture input, which is desirable for data augmentation.

We validated the proposed method using a car inspection dataset that provides approximately 8000 pairs of semantic mask labels and BSX images for training. In addition to qualitatively comparing the generated images, we measured the quantitative performance of the proposed method by comparing both the Fréchet inception distance (FID) and the accuracies of the semantic segmentation of car parts, car model type classification, and car detection. To ensure that the comparison was fair, the networks for each task were trained using the samples generated by the competing methods and then tested on real BSX car images. We tested various texture inputs computed using the Sobel edge filter (SEF) [14], local binary patterns (LBP) [15], and local thresholding (LTH) [16]; and we confirmed that LBP and LTH were highly effective.

The remainder of this paper is structured as follows: Section 2 briefly reviews related works, Section 3 introduces the proposed method, Section 4 provides the experimental results, and Section 5 describes the conclusions.

## 2. Related Work

### 2.1. Automated Container Inspection

Among cargo items, detecting vehicles in a container X-ray image is one of main subjects that has attracted the attention of both researchers and customs. This study aimed to develop an automated X-ray inspection system to detect vehicles quickly declared in customs. Falsely reported vehicles are those involved in crimes such as theft and are being smuggled overseas [17,18]. As these smuggled vehicles are used for criminal gang and terrorist activities, it is important for customs to detect them. Early stage studies for vehicles [11,19] were carried out by Jaccard et al. In [19], sub-windows of container X-ray images were classified as "car" and "non-car" using a random forest classifier and feature histogram. As a result of the experiment, there was a 100% detection rate and there were 1.23% false positives. In their follow-up study [11], CNN was applied for vehicle detection. The number of samples was increased by oversampling the training data: 100% detection rate and 0.22% false-positive rate were calculated. It has also been confirmed that vehicles that are heavily obscured can be detected.

In addition to vehicles, studies have been conducted to automate X-ray inspection of general cargo items. Tuszynski et al. [20] proposed a method to classify cargo items by calculating the image feature distance between new cargo items and registered items. Using this method, 31% of items were classified into the correct category, and a top-5 accuracy of 65% was obtained. Zhang et al. [21] proposed a cargo X-ray classification method using joint shape and texture features. The best classification performance was obtained when the SVM classifier with joint shape and texture features was used, but the accuracy for the 22 classes was 55%.

### 2.2. Data Augmentation Using a Generative Adversarial Network

Early studies on X-ray cargo inspection mention the lack of data as a challenge. In these studies, to solve the data shortage issue, objects are cropped and then data synthesized by projecting them onto a container [5,6,7,8,9,10]; classical techniques such as oversampling [11] are also used. However, existing methods have limitations, as they cannot increase the diversity of objects to be detected. To overcome these limitations, the data augmentation method based on a generative adversarial network (GAN) has been used in various fields of research [22,23,24,25].

From this perspective, we can examine the evolution of GAN-based image-to-image translation techniques, which map an input image to a corresponding output image. Pix2pix [13] is a seminal method for image-to-image translation tasks with paired training data. We propose a typical method using a “U-net”-based architecture as the generator and “PatchGAN” classifier as the discriminator. In tasks with unpaired training data, CycleGAN [26] showed that a cycle consistency constraint can help to learn two different unpaired data distributions. CD-GAN [27] and C-CycleGAN [28] showed that control parameters called conditions can also be embedded into GANs for both paired and unpaired cases, respectively. TextureGAN [29] first used the texture patch directly extracted from the target image as a control parameter. Its generative network learns to synthesize objects consistent with the texture patch. To the best of our knowledge, however, they authors presented only qualitative results, and the effects of generator inputs on GAN were not tested. StyleGAN [30] introduced the intermediate latent space W, which controls the generator and used the adaptive instance normalization to disentangle styles and semantics properly, and thereby enhance details. However, the latent space determination is implicit and complex. SPADE [31] proposed a method to solve the issue of losing semantic information of the image generation process using spatially-adaptive normalization layers. On the other hand, there have been studies trying to reduce the structural instability of GAN training, by introducing losses based on more sophisticated distribution similarity measures such as WGAN [32], or by employing a different training strategy based on a game theory framework, as proposed in [33]. Although these studies show improved performance in image generation, these techniques are rarely employed in image translation. Training a network using the original GAN loss is much more straightforward in image translation due to the pixel-wise loss from ground-truth images, which prevents training from being stuck in undesired local minima.

The study on GAN was extended to research on domain randomization and domain adaptation (e.g., [34]). Research areas such as heterogeneous face recognition (HFR) and computer-aided diagnosis (CAD) have also benefited from GAN. For instance, image conversion techniques to convert thermal images into visible images were introduced in [35,36]. Additionally, detecting abnormal objects in medical images was addressed in [37]. In such problems, synthesizing natural fake images while maintaining identity is an important issue. To resolve this issue, research on image composition ranging from classical approaches, such as Poisson blending [38], to recent approaches, such as deep image harmonization [39], has been increasing. In addition to the aforementioned studies (e.g., [11,19]), research to detect dangerous objects in X-ray images of small luggage was recently introduced (e.g., [23,40]). However, their findings were not applicable to problems involving severe noises that can be seen in BSX images and the difficulty of synthesizing images by simply using simple blending techniques.

Recently, studies such as [41,42] showed that GAN image generators easily fail to approximate the spectral distributions of real data. In this paper, we propose a texture parameterized generative network to mitigate these problems and apply to real cases, such as cargo inspection using BSX images.

## 3. Method

### 3.1. Proposed Data Augmentation and Inspection Process

**Figure 1:**
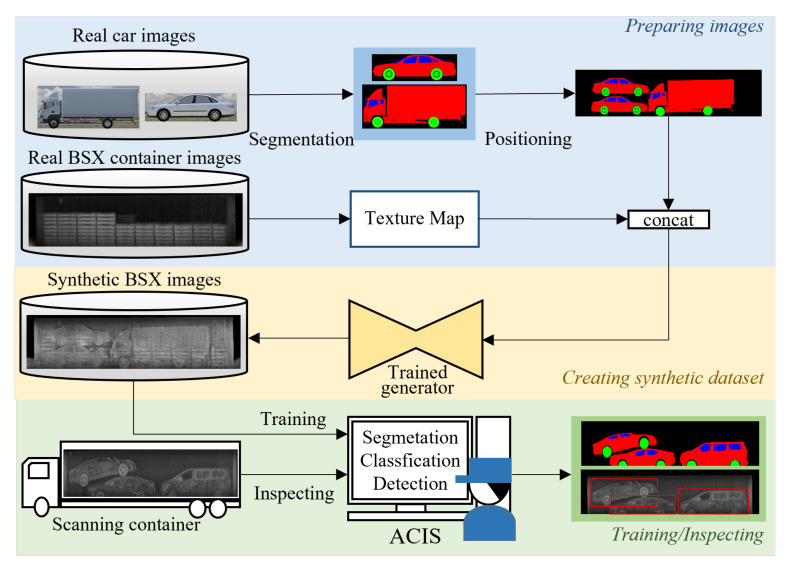
Overall structure of the automatic cargo inspection process (ACIS) using the proposed BSX image generation method.

Figure 1 illustrates our automatic cargo inspection system (ACIS) equipped with the proposed GAN network. In the stage of preparing images, we collected real images of custom items, cars in this case, which are renewed every year, and then segmented them into three classes, body, window, and wheel. Just like shipping cars in containers, the segmentation masks of cars are plausibly packed in a container-sized mask. This process can be fully automated. In the stage of creating synthetic images for data augmentation, we composited the container-sized segmentation masks and the real BSX container images using the proposed GAN network, which was trained by a few real BSX scanned images. The resulting synthetic BSX images can be used as training data for automatic cargo inspection system development.

### 3.2. Proposed GAN Network

Conditional GAN methods, such as pix2pix, take a condition *x* as an input of the generator *G* along with latent noise *z* and produce a corresponding fake image G(x,z). The discriminator *D* is learned to discriminate a ground truth image *y* from the generated image. Overall, The image generation process of pix2pix [13] is summarized by Equations (1)–(3).

(1) $$\begin{array}{cc} G^{★} & =\underset{G}{\mathrm{argmin}}\;\max_{D}\left(L_{cGAN}\left(G,D\right)+\lambda L_{1}\left(G\right)\right) \end{array}$$

(2) $$\begin{array}{cc} L_{cGAN}\left(G,D\right) & =E_{x,y}\left[\log D\left(x,y\right)\right]+E_{x,z}[\log\left(1-D\left(x,G\left(x,z\right)\right)\right] \end{array}$$

(3) $$\begin{array}{cc} L_{1}\left(G\right) & =E_{x,y,z}{[∥y-G\left(x,z\right)∥}_{1}] \end{array}$$

where λ is a balancing parameter to control the significance of the reconstruction loss (3) relative to the adversarial loss (2). The value of λ was set to 100, which was empirically chosen, throughout the paper. In the objective function (2), *D* tries to maximize this objective, and *G* tries to generate images like the real distribution as much as possible to reduce the loss against *D*. $E_{x,y}$ and $E_{x,z}$ are the expected values over all real data *y* and generated data G(x,z) with given condition *y*.

The reconstruction loss (3) is calculated by averaging the color differences between the ground-truth image and generated image. The sole use of reconstruction loss results in oversmoothed images due to its averaging effect. Hence, it requires the adversarial loss (2), which makes an overall look of the generated image as similar as possible to that of the given image, so that the small details and textures can be reproduced.

Even if we use adversarial loss, however, the visual gap between the real and generated images is still substantial; fake BSX images generated by pix2pix are blurrier than the real BSX images. The situation worsens if we synthesize an image by placing the generated image patches on a background container image to use for data augmentation of object detection or semantic segmentation. In such cases, it is not straightforward to process the boundaries of the overlaid object patches, since the unnatural borderlines between images might cause the trained model to be overfitted in an undesirable way. An image blending method, such as the Poisson image blending, can be used for more natural synthesis. In our experiments, however, some visually important features, such as object outlines of the generated images, are lost during the blending process.

To remedy the problem that adversarial loss used in pix2pix failed to reproduce the BSX-specific noise pattern, we conjectured that an indicator that can measure and quantify texture can help with texture creation. As indicators, we tested a Sobel edge filter (SEF) [14], local binary patterns (LBP) [15], and local thresholding (LTH) [16]; and found they can effectively disclose the difference of texture information between the generated images and real BSX images. To use these texture metrics, we added a generalized texture map *t* as an input to the generator. Then, the proposed process replaces Equations (2) and (3) with Equations (4) and (5).

(4) $$\begin{array}{cc} L_{cGAN}\left(G,D\right) & =E_{x,y}\left[\log D\left(x,y\right)\right]+E_{x,z,t}[\log\left(1-D\left(x,G\left(x,t,z\right)\right)\right] \end{array}$$

(5) $$\begin{array}{cc} L_{1}\left(G\right) & =E_{x,y,z,t}{[∥y-G\left(x,t,z\right)∥}_{1}] \end{array}$$

(6) $$\begin{array}{cc} t & =\left\{\begin{array}{c} \mathrm{ExtractTexture}(y)\;\mathrm{for}\;\mathrm{training}\\ \mathrm{ExtractTexture}(b)\;\mathrm{for}\;\mathrm{testing} \end{array}\right. \end{array}$$

where *ExtractTexture* represents the process to extract texture map *t* from the BSX image. Texture map *t* is extracted from target *y* during training, as shown in (6), and after training, a new image is created by extracting a texture from a random background *b*.

Improving the resolution by adding information to the input has also been attempted with pix2pixHD [43], where boundary information is added to the input to sharpen the boundary between objects. However, our method attempts to reproduce domain-specific image characteristics by explicitly feeding a texture map into the generator.

**Figure 2:**
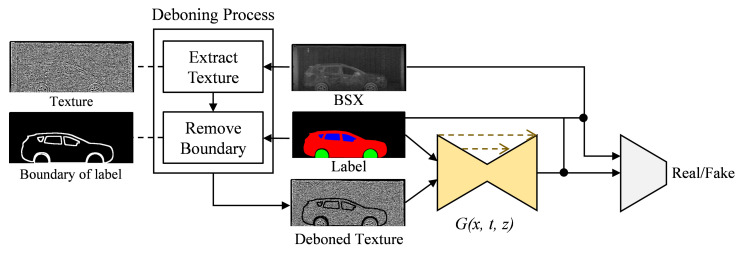
Proposed method to train the generative adversarial network by using the BSX texture image as an input.

The overall architecture of the proposed method is drawn in Figure 2 and the training and inference steps are summarized as follows:

**Training:**

**Input:**  BSX-car images and their paired labels
**Step1:**  Extract texture maps from BSX-car images using one of (7), (10) and (15).
**Step2:**  Remove object’s boundaries from the texture maps using the labels.
**Step3:**  Train the networks using the texture maps and the pairs of images and labels.

**Inference:**

**Input:**  Arbitrary BSX-background images and labels
**Step1:**  Extract texture maps from BSX-background images using one of (7), (10) and (15) used for training.
**Step2:**  Remove object’s boundaries from the texture maps using the labels.
**Step3:**  Generate synthetic BSX images from the trained generator with the texture maps and the labels as inputs.

Texture extraction is described in detail as follows: the SEF [14] uses the second derivative and is formulated using (7)–(9). In the second derivative, the gradient is large at the edge where the brightness changes; the edge is detected in this way. The texture map extracted with SEF as input was used to generate mammograms [44]. Since the second derivative is sensitive to noise, it can be assumed that it is one of the features that can express noise well.

(7) $$G=\sqrt{G_{w}^{2}+G_{h}^{2}},$$

(8) $$G_{w}=\left[\begin{array}{ccc} +1 & 0 & -1\\ +2 & 0 & -2\\ +1 & 0 & -1 \end{array}\right]⊛I,$$

(9) $$G_{h}=\left[\begin{array}{ccc} +1 & +2 & +1\\ 0 & 0 & 0\\ -1 & -2 & -1 \end{array}\right]⊛I,$$

where *G* is the magnitude of Sobel edge, and $G_{w}$ and $G_{h}$ are the gradients of image *I* in the *w* and *h* directions, respectively.

LBP [15] is a feature developed to classify textures. It is a value calculated for all pixels of an image and an index value obtained by coding the change in brightness of the surrounding area of each pixel in binary. Textures (residue, forest, land, etc.) are classified using methods such as histogram matching for index values [45,46]. LBP is formulated as (10)–(14).

(10) $$L_{p,R}\left(r_{c},c_{c}\right)=\sum_{p=0}^{P-1}s\left(g_{p}-g_{c}\right)2^{p},$$

(11) $$s\left(q\right)=\left\{\begin{array}{cc} 1, & q\geq 0\\ 0, & \mathrm{otherwise} \end{array}\right.,$$

(12) $$g_{p}=I\left(r_{p},c_{p}\right),p=0,\ldots ,P-1,$$

(13) $$r_{p}=r_{c}-R\sin\left(2\pi p/P\right),$$

(14) $$c_{p}=c_{c}+R\cos\left(2\pi p/P\right),$$

where *L* is the image intensity, $g_{p}$ represents the grayscale values of the *P* sample points $r_{p}$ and $c_{p}$ among pixels located around the radius *R* at the center coordinates $r_{c}$ and $c_{c}$.

LTH [16] is usually used to separate objects and backgrounds based on the difference in contrast. However, it can also be used as a method for expressing textures. Thresholding has been used for microstructure evaluation and quantitative analysis of texture characteristics by applying the morphology technique to the thresholded images [47]. Therefore, thresholding images is considered one of the methods to characterize texture well. LTH is formulated as (15) and (16).

(15) $$T\left(r_{c},c_{c}\right)=\left\{\begin{array}{cc} 1, & I\left(r_{c},c_{c}\right)\geq m\left(r_{c},c_{c}\right)\\ 0, & \mathrm{otherwise} \end{array}\right.,$$

(16) $$m\left(r_{c},c_{c}\right)=\frac{1}{k^{2}}\sum_{i=c_{c}-\left\lfloor \frac{k}{2}\right\rfloor }^{c_{c}+\left\lceil \frac{k}{2}\right\rceil }\sum_{j=r_{c}-\left\lfloor \frac{k}{2}\right\rfloor }^{r_{c}+\left\lceil \frac{k}{2}\right\rceil }I\left(i,j\right),$$

where *T* is the binary image, *k* is the kernel size, and $r_{c}$ and $c_{c}$ are the center coordinates. In the experiments, *R* and *k* were set to 3, which is the smallest value that can be set. The smaller the value, the denser texture can be extracted. *P* is the number of sample points, and was set to 8, which is appropriate for *R* = 3.

**Figure 3:**
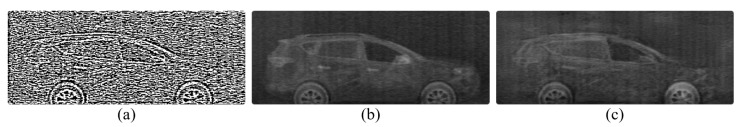
Image translation results when only the texture input was fed into the generator. The texture input (**a**) was extracted from a real BSX image (**b**), and the result was (**c**).

The deboning process is a newly introduced important process to consider when using texture maps. If we feed texture images extracted from real BSX images to the generator during training, the network learns to restore a BSX image from the input texture image without reference to the semantic label map, as shown in Figure 3. The network was given only the texture map (Figure 3a) as input by setting all the pixel values of the label map to zero. The generated image in Figure 3c is almost the same as the real BSX image, Figure 3b, which implies that the generator is trained to extract shape information of the car and texture information from the texture input, ignoring the semantic label.

However, the goal of the network is to translate the semantic label into a BSX image with the aid of texture input reproducing BSX-specific image characteristics. To exclude this trivial solution, we propose a deboning process. The boundary is extracted using the difference between the dilation and erosion of the label; the texture map boundary is removed by approximately seven pixels for the experiments.

**Figure 4:**
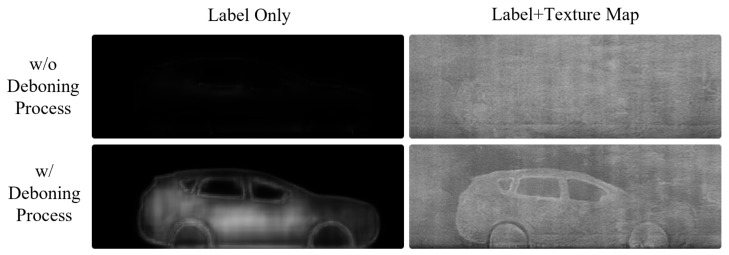
The effect of the deboning process is qualitatively described. The top row shows that when the full texture input is fed during training, the generator ignores the information in the label map completely. Application of the deboning process allows the model to use the shape information from the label map, and the texture map is used to reproduce background information and BSX-specific noise patterns.

The top images of Figure 4 show the inference results of the generator trained without the deboning process. In the top-right image in Figure 4, the car shape does not appear although the provided semantic label map contains the shape information. The top left figure, generated by using the semantic label only, also confirms that the generator was trained to ignore the mapping between car shapes and semantic labels. Conversely, the bottom row in Figure 4 shows that a simple technique for removing the boundary plays an important role in allowing the generator to learn the car shape from the label. The car shape is successfully restored, even when the semantic label is solely used. As a result, a more natural and realistic synthetic BSX image can be generated when the network is trained with a boundary-removed texture map.

### 3.3. Dataset Construction

**Figure 5:**
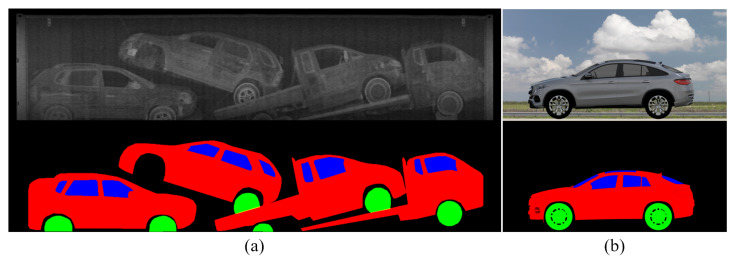
Sample images of datasets: (**a**) Real BSX image and the corresponding segmentation label from the BSX-car dataset. (**b**) Rendered image of a 3D model and the corresponding segmentation label from the 3D-car dataset.

**BSX-car dataset**: We collected real BSX scanned images with the cooperation of the national customs service to compose a dataset to train and test the proposed method. The number of scanned images was 1776. We sampled 1136 images for training, 284 images for testing, and used the remainder for validation. The BSX images were annotated by human operators to produce (1) semantic label images with three semantic classes, i.e., body, window, and wheel; (2) bounding boxes of car instances; and (3) car class labels for each bounding box. Figure 5a shows a sample from the training data with ground-truth annotations. Each scanned image contains five cars on average; hence, we could obtain approximately 8000 BSX car images.

**3D-car dataset**: In a real ACIS application scenario, it is not always possible to have BSX scanned images for a specific car model. Thus, we constructed approximately 1000 3D car models. As there is no way to render a 3D model into a BSX image, the dataset cannot be used for training; only the semantic label images were used during the test phase. Figure 5b shows a 2D rendering result of a 3D model with corresponding segmentation masks.

## 4. Experiments

In the following subsections, we summarize the experimental results of the proposed method obtained using various texture parameters, namely, SEF, LBP, and LTH. The proposed method and pix2pix are trained by using only the training data from BSX-car dataset. They were trained using the same experimental settings except for the presence of additional texture parameter input. The resolution of the input semantic label, texture parameters, and the output BSX image was 300 × 1200 pixels; the networks were trained for 300 epochs with eight samples per batch. We first compared the visual quality of the fake images synthesized by the proposed method and the vanilla pix2pix. Then, we quantitatively evaluated the proposed method by measuring the performance improvement achieved when using fake images for data augmentation on various computer vision tasks, including segmentation, classification, and detection.

### 4.1. Qualitative Evaluation

**Figure 6:**
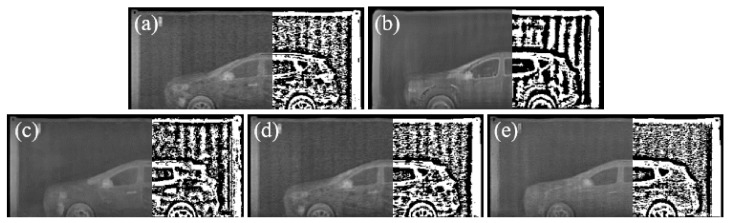
Qualitative comparison of texture patterns: (**a**) Real BSX image. (**b**) Result of pix2pix. (**c**–**e**) Results of the proposed method based on SEF, LBP, and LTH, respectively.

**Figure 7:**
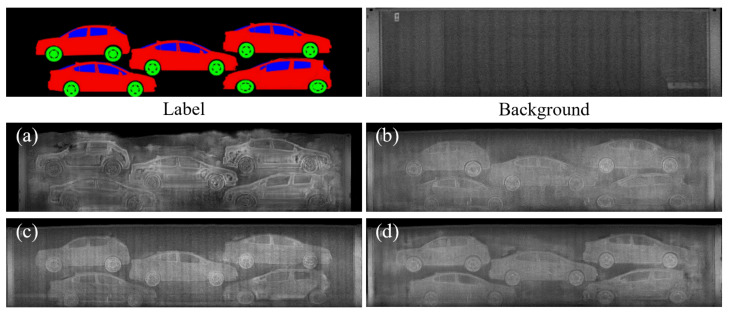
Qualitative comparison of image synthesis: semantic label and background (top row) are inputs to generate the synthetic images of the proposed method. (**a**) Result of pix2pix. (**b**–**d**) Results of the proposed method based on SEF, LBP, and LTH, respectively.

For the qualitative comparison, Figure 6 and Figure 7 show the image translation and synthesis results obtained using vanilla pix2pix and the proposed methods with various texture inputs. The texture map used to generate Figure 6 was extracted from a real BSX image. In Figure 6, although the generated images depict the vehicle’s shape well, the image generated with pix2pix is slightly blurred. Since it is difficult to distinguish such a texture difference in the image, local thresholding of the image is shown on the right part of the image to clearly visualize the difference in texture. It can be seen that the objects (white area) extracted through local thresholding are coarser in Figure 6b than in Figure 6a. In contrast, in the images (Figure 6c–e) using the texture map, the texture appears to be fine, and the texture of the image using LBP and LTH is more similar to that of the real BSX image.

Figure 7 shows the image generated using the texture map extracted from the arbitrary container, the top-right of Figure 7, and the label map, top-left of Figure 7. The images from the vanilla pix2pix appear oversmoothed except for the object boundaries, which are overexaggerated, and there are severe artifacts around the borders of the containers. We attribute this to the failure of GAN in reconstructing various container shapes and sample-specific BSX noise patterns, which largely vary in the presence of background clutter, given a clean semantic label image as the only input. In contrast, the images obtained by the proposed method show more realistic texture patterns with fewer undesired artifacts: especially around the borders of the containers, with the aid of background information, which is not obtainable from semantic label images.

**Figure 8:**
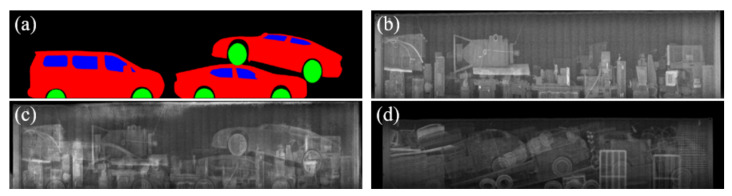
Synthetic BSX image generated by the proposed method with occluding objects in the background. (**a**) Semantic label image. (**b**) Background image to compute the texture parameters for the proposed method. (**c**) Results of the proposed method based on LTH. (**d**) A real BSX image with severe occlusion for reference.

**Figure 9:**
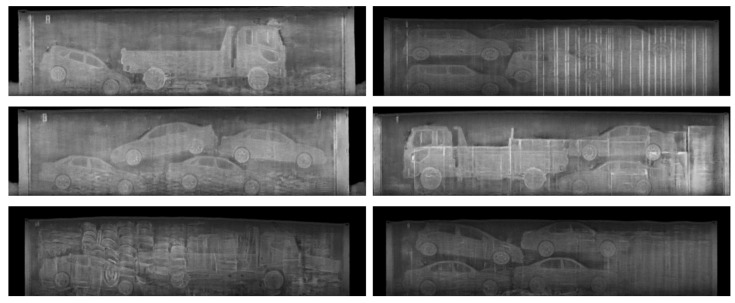
Synthetic BSX image samples. All were generated by the proposed method using texture map LTH and semantic labels segmented from 3D cars and positioned using a packing algorithm.

Another appealing advantage of using the texture input, in terms of data augmentation, is that the quantity and quality of the synthesized images, can be easily increased by adding more background images without additional manual intervention for labeling. Figure 8 shows that the proposed method can generate various synthetic images with one semantic label template by feeding various background texture inputs. Moreover, the proposed method can successfully reproduce occlusions and overlaps among objects utilizing cluttered background textures. Considering the frequent observations of these patterns in real BSX images, the capability of reproducing these characteristics is crucial to narrowing the differences between the distributions of the real data and the synthetic data. Figure 9 shows more synthetic BSX images generated by the proposed method using a texture map LTH and semantic labels segmented from 3D-cars and positioned using packing algorithm. In this way, we can obtain high quality training data for unseen objects.

### 4.2. Fréchet Inception Distance (FID)

The visual quality of the generated image is typically measured to evaluate the performances of different GAN models or parameters. The FID [48] is used to quantitatively measure the visual quality of the generated image and indicates the similarity between two image datasets. The FID is the Fréchet distance between feature vectors extracted using the inception network and is calculated as

(17) $$\begin{array}{cc} d^{2}\left(\left(\mu_{r},C_{r}\right),\left(\mu_{g},C_{g}\right)\right) & =∥\mu_{r}-\mu_{g}{∥}_{2}^{2}+\mathrm{Tr}\left(C_{r}+C_{g}-2\sqrt{C_{r}C_{g}}\right) \end{array}$$

where the subscripts *r* and *g* refer to the real and generated images; and μ and *C* are the feature-wise mean and the covariance matrix. Tr refers to the trace linear algebra operation.

Another widely used measure to assess the quality of the generated images is sliced Wasserstein distance (SWD) [49], which approximates earthmover’s distance in a computationally efficient manner. In this paper, we followed the same computational procedure to compute SWD as in [49]. For both FID and SWD, the lower score represents good quality.

Table 1 shows the FID and SWD scores according to the texture map. The FID scores of LBP and LTH were 27.6 and 28.1, respectively, which were lower than that of pix2pix, which was 43.9 without a texture map. The tendency is consistent when the SWD scores are compared. We also tested the generator containing residual blocks used in [26] and the SPADE generator introduced in [31], but the generator using U-net of pix2pix showed the best result. This shows that our choice of pix2pix as the base model is effective and the tendency is similar in the test results in terms of data augmentation, which is explained in Section 4.3, Section 4.4 and Section 4.5. Additionally, comparative experiments with SPADE were added to Appendix B.

The FID and SWD results indicate that by using the appropriate texture features as parameters, the distribution of generated images approaches that of the real BSX images, enabling effective data.

### 4.3. Segmentation

The target task in this experiment was to estimate a semantic label image of car parts from a BSX image, which is a backward operation of the image-to-image translation. To estimate the effectiveness of the fake images in terms of data augmentation, we trained the segmentation network [50] with different training data combinations and measured the performance on the test set of the BSX-car dataset. The performance was measured in terms of pixelwise accuracy and the mean intersection of union (mIoU). In the following, $L_{3D}$ and $L_{BSX}$ denote fake images generated from the labels of the 3D-car dataset and BSX-car dataset, respectively.

**Figure 10:**
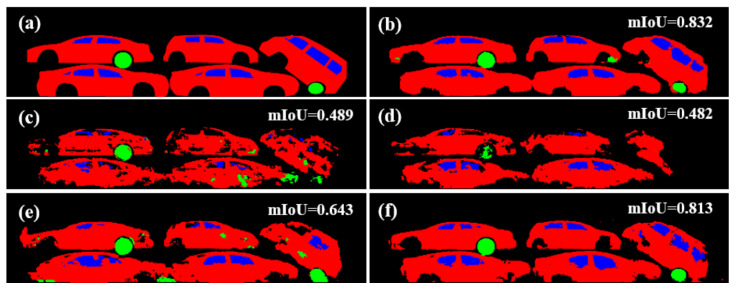
Segmentation results when different fake images are used as the training set: (**a**) Ground-truth semantic label. (**b**) Result obtained by using real BSX training data. (**c**–**f**) Results obtained by using only fake images from pix2pix, SEF, LBP, and LTH, respectively.

Table 2 summarizes the results when the network was trained only using the fake images and using both fake and real images. The top row shows the baseline results, for which the real BSX image and semantic label pairs from the BSX-car dataset were used for training. Using a texture parameter consistently improved the quality of the images synthesized with pix2pix, as qualitatively seen in Figure 10. The gap between the baseline and each result indicates that the images generated by the proposed method are closer to the real BSX images than those of the baseline.

It is noteworthy that the use of semantic label images from the BSX-car dataset resulted in a better performance than the use of the 3D-car dataset. This result was due to a little unnatural arrangement of the car semantic labels in the 3D-car dataset to create a larger container semantic label image, resembling the semantic label image of a real BSX container image. Although we exploited the packing algorithm [51] to synthesize realistic label images by stacking the cars with a random rotation and flip in a nonoverlapping manner, there should be inevitable differences between the real semantic label images and the synthesized semantic label images.

In contrast, when the fake images were used along with the real BSX training data, the use of $L_{3D}$ showed better results than $L_{BSX}$, as shown from the second to fifth row in Table 2. In this case, the differences in the synthesized label images in $L_{3D}$ from the real semantic label images help to generalize the trained network. In fact, the semantic label images used to generate fake images from $L_{BSX}$ are the same as those in the real training data, making the use of the fake images from $L_{BSX}$ relatively redundant. Nonetheless, the use of the fake images from $L_{BSX}$ made by various random textures or even the oversmoothed fake images from pix2pix improved the baseline training data, which supports the use of GAN for data augmentation when the quantity of the data is insufficient.

**Figure 11:**
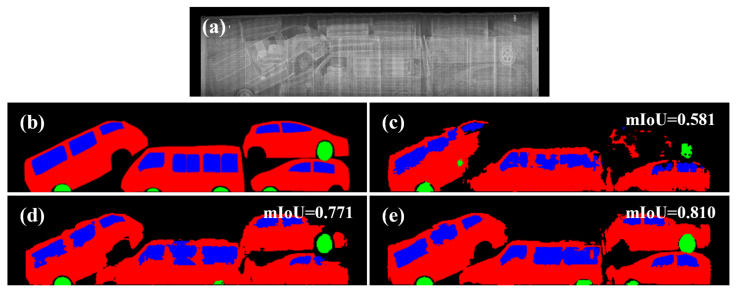
Segmentation results for a sample with severe occlusion: (**a**) Input BSX image. (**b**) Ground-truth semantic label. (**c**) Result obtained by using real BSX training data. (**d**,**e**) Augmentation results using fake images generated by pix2pix and LTH, respectively.

In both experiments, the use of fake images using LTH attained the overall best performance. Figure 11 visualizes the segmentation results of a test sample with a low mIoU value, even if the model learned from real data. We can see the effectiveness of our method is due to the ability to depict realistic occlusion.

**Figure 12:**
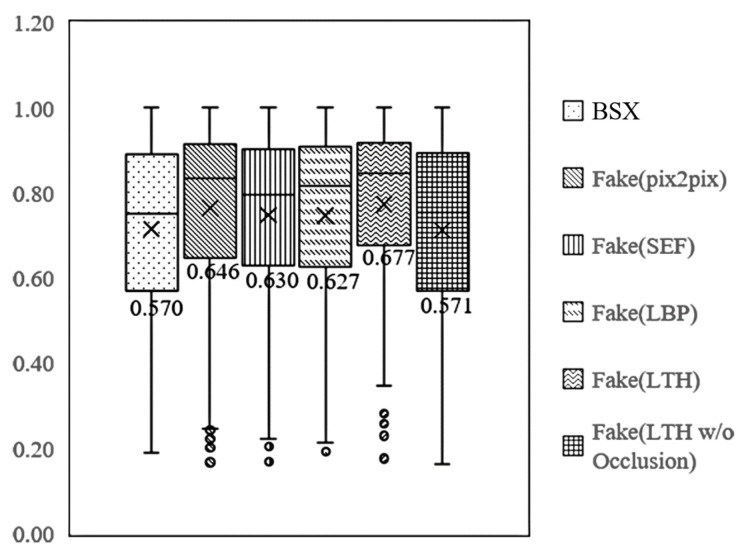
Distribution of mIoU of segmentation according to the texture map.

Figure 12 shows the distribution of mIoU according to the dataset. Looking at the lower quartile (Q1) and minimum values, the Q1 of the real BSX data and fake data with the LTH textures are 0.570 and 0.677, respectively, and the difference is 0.107. An increase in Q1 indicates that the proportion of segmentation results for the lower 25% decreased. In other words, the segmentation performance for BSX, where mIoU was in the lower 25%, was significantly improved. Figure 11a is an example image corresponding to the value of 0.570, which is the Q1 of real BSX data. The figure shows an image in which occlusion is present; mIoU is greatly improved using fake data, particularly with the LTH texture. In the case of a background container image without occlusion, the Q1 of fake data was 0.571, which indicates that there was no improvement. Therefore, the proposed method improved the segmentation performance because of the effect of depicting occlusion. If there is no occlusion, then fake images generated using only the label can improve performance.

### 4.4. Classification

The next task was to classify a BSX-car image. We first cropped the original BSX container images to produce smaller image patches, 224 × 512, each containing a single car, based on the ground-truth bounding box information. After filtering out the images without car model annotations, we obtained 732 BSX-car images with 11 car models as shown in Appendix A. Among them, 590 images were used for training, and 142 images were used for testing.

The number of fake images generated for data augmentation was 200 per class. Unlike the other tasks, we randomly removed wheels in the input label images during the image generation to reflect the fact that wheels are frequently missing in real BSX images. At each training iteration, we randomly sampled fake images to compose a batch in which the ratio of real BSX samples and fake image samples was kept at 1:1. The ResNet50 architecture [52] was used for experimentation, and the networks were trained for 1000 epochs.

The use of fake images made by the LTH texture and $L_{3D}$ showed the best improvement, as summarized in Table 3, achieving an approximately 6.3% increase in classification accuracy. In contrast, the fake images generated using $L_{BSX}$ showed less improvements, and the accuracy even decreased in the case of pix2pix and SEF. We speculate that the texture details of the fake images are more important for effectiveness in data augmentation, for classification tasks.

**Figure 13:**
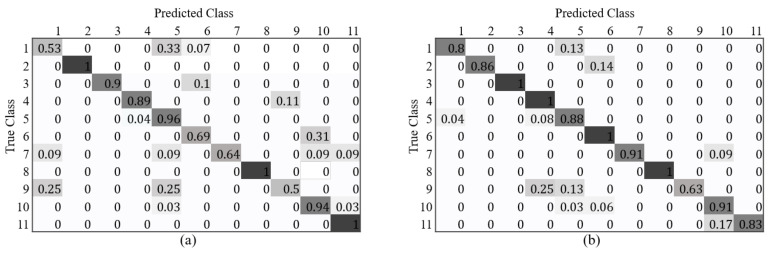
Confusion matrix of classification accuracy according to training set: (**a**) BSX only; (**b**) BSX + Fake (LTH) with $L_{3D}$.

**Figure 14:**
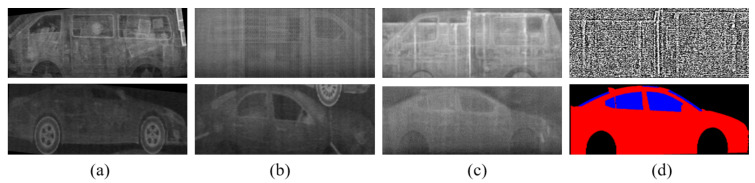
The classification method trained with only the real BSX images can correctly classify samples in (**a**), but failed to estimate the class labels for samples in (**b**), which are correctly classified after data augmentation. Column (**c**) shows synthetic images generated by using the proposed method with edited inputs shown in (**d**) during data augmentation. The top and bottom samples belong to class 6 (the texture map is edited by drawing random white lines to simulate severely occluded samples) and class 1 (the label image is edited to omit the wheels), respectively.

Figure 13 shows the confusion matrices of the classification accuracy for each vehicle class according to the BSX and BSX + Fake (LTH) training datasets. When training with BSX + Fake (LTH), the accuracy of some vehicle models decreased slightly, but overall accuracy improved. The large improvements in classification accuracy for class 1 and 6 are noteworthy. We attribute this to the effect of a deliberately designed data augmentation scheme, where the generated images are supposed to have many severely occluded cars or cars without wheels to better imitate the real BSX images. The ratio of severely occluded cars is relatively larger for class 6, and many car images are without wheels for class 1, as shown in Figure 14. The capability of editing the texture map and the input label image for data generation is one of the advantages of the proposed method.

### 4.5. Detection

We tested the validity of data augmentation using fake images for object detection. In this experiment, we classified cars into five broad categories: hatchback, sedan, SUV, truck, and van. Among the 1776 images of the real BSX dataset, only 1026 images contained the corresponding car instances; hence, we used 814 images for training and 212 images for testing. A total of 1758 fake images were generated to augment the real data, and they were randomly sampled at each training iteration to make the ratio of real data and fake data 1:1. For each training data configuration, we trained a RetinaNet [53] for 30 epochs.

**Figure 15:**
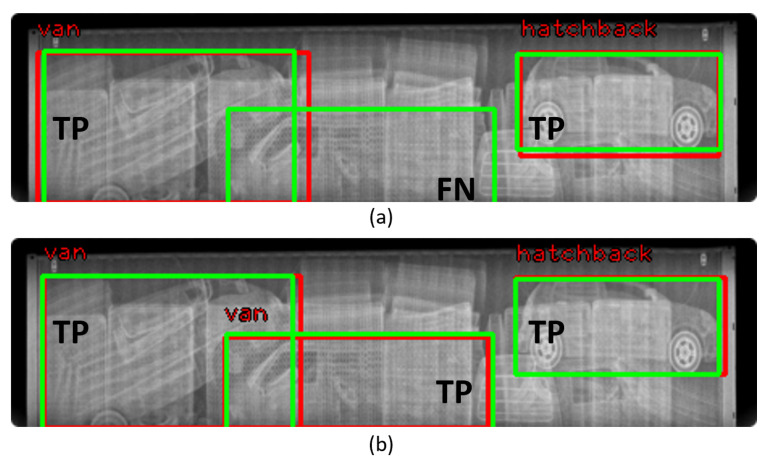
Comparison of detection results: (**a**) result of training with real BSX data; (**b**) result of training with Real+Fake (LTH). Green: ground truth; Red: prediction. TP: true positive; FN: false negative.

Figure 15 shows the detection result: (a) and (b) are the results of inference according to training with real and real+fake (LTH), respectively. As shown in Figure 15a, the vehicle located in the middle of the image could not be detected, but in Figure 15b, it can be confirmed that the vehicle was accurately detected. This shows that real+fake (LTH) contributed to the detection of occluded vehicles.

Table 4 shows the quantitative results. The use of fake images made using the LBP texture produce the best results, with a maximum accuracy gain of 1.7%. It is noteworthy that the detector learned only from fake data had a small difference, 0.2%, in performance from the detector learned from real data.

## 5. Conclusions

In attempts to apply GAN-based image-to-image translation techniques to actual recognition problems, it has been reported that GAN cannot approximate all spectrum of real data distribution. In this paper, we presented a simple and effective method for translating a semantic label into a realistic image and overcoming the limitations. We applied it to deep learning-based automatic cargo inspection.

The proposed method uses a texture map obtained from the deboning process along with the semantic label to generate unique textures in the BSX image. The texture maps, such as a Sobel edge filter, local thresholding, and local binary pattern, were tested; and the characteristics of the generated images according to each texture map were compared. The images generated using the LBP and LTH exhibited natural background textures, in contrast to the blurry results generated using only the semantic label, as in the previous method. In addition, using the texture map, the occlusion phenomenon that exists in the actual container image can be successfully reproduced.

We also performed the segmentation, classification, and detection of BSX images, which are less researched than transmissive X-rays. The experiments showed that the use of images generated by the proposed method to augment the real training data consistently improved the performance of the baseline network for each problem. The results of this study can be used as a reference not only in future studies using X-ray images but also in studies using ultrasound or synthetic-aperture radar (SAR) images that have scattering noise.

## Figures and Tables

**Table 1:** FID and SWD of images generated according to the texture map.

Generated Image	FID	SWD
Fake (pix2pix)	43.9	1393.97
Fake (SEF)	42.1	1283.53
Fake (LTH)	28.1	1133.91
Fake (LBP)	**27.6**	**892.97**

**Table 2:** Effect of fake images on the segmentation performance.

	Accuracy		mIoU	
Training Dataset	$L_{3D}$	$L_{\mathrm{BSX}}$	$L_{3D}$	$L_{\mathrm{BSX}}$
Real	0.909		0.715	
Real + Fake (pix2pix)	0.921	0.915	0.764	0.726
Real + Fake (SEF)	0.914	0.914	0.748	0.739
Real + Fake (LTH)	**0.925**	**0.921**	**0.773**	0.754
Real + Fake (LBP)	0.920	0.918	0.746	**0.757**
Fake(pix2pix)	0.677	0.793	0.250	0.468
Fake(SEF)	0.708	0.798	0.293	0.489
Fake(LTH)	**0.772**	**0.867**	**0.407**	**0.621**
Fake(LBP)	0.748	0.839	0.395	0.563

**Table 3:** Classification accuracy according to the training data.

	Accuracy	
Training Dataset	$L_{3D}$	$L_{\mathrm{BSX}}$
Real	0.838	
Real+Fake(pix2pix)	0.866	0.810
Real+Fake(SEF)	0.873	0.775
Real+Fake(LTH)	**0.901**	**0.859**
Real+Fake(LBP)	0.866	0.838

**Table 4:** Detection accuracy according to the training data.

	Accuracy	
Training Dataset	$L_{3D}$	$L_{\mathrm{BSX}}$
Real	0.899	
Real+Fake(pix2pix)	0.907	0.911
Real+Fake(SEF)	0.897	0.913
Real+Fake(LTH)	0.912	0.910
Real+Fake(LBP)	**0.913**	**0.916**
Fake(pix2pix)	0.174	0.804
Fake(SEF)	0.247	0.825
Fake(LTH)	0.248	0.889
Fake(LBP)	**0.298**	**0.897**

**Figure A1:**
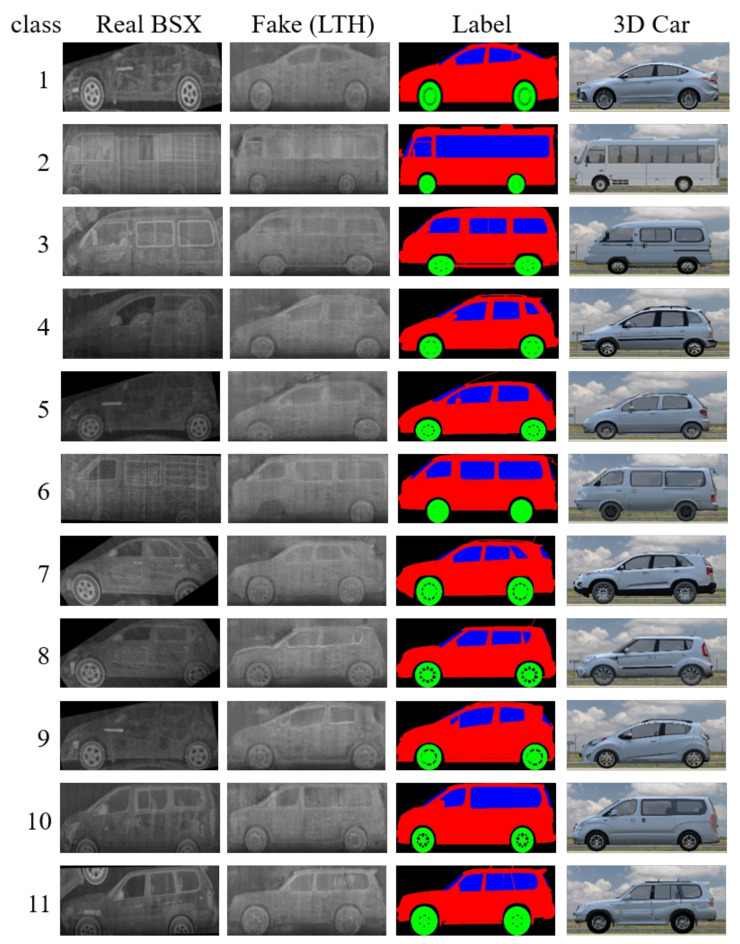
Real and Fake (LTH) BSX images used for classification. Fake images were generated from the semantic labels of the 3D cars. class 1: AVANTE, 2: BUS, 3: DAMAS, 4: LAVITA, 5: MATIZ, 6: PREGIO, 7: SORENTO, 8: SOUL, 9: SPARK, 10: STAREX, 11: TERRACAN.

## Data Availability

Data used in this study can be made available upon reasonable request.

## Abbreviations

The following abbreviations are used in this manuscript:

GAN	Generative adversarial network
BSX	Backscatter X-ray
ZBV	Z-backscatter van
FID	Fréchet inception distance
SEF	Sobel edge filter
LBP	Local binary patterns
LTH	Local thresholding

## Appendix A

Figure A1 shows the 11 vehicle images used in the classification experiment, their model names, and 3D cars and their labels.

## Appendix B

A comparison with SPADE [31], one of the state-of-the-art image translation methods, has been performed. Due to the large memory requirement of SPADE, we reduced the size of test images from 300 × 1200 to 128 × 512 for this experiment. Table A1 shows the FID measurement results. Note that, unlike the label-to-RGB image translation, the pix2pix showed better performance than SPADE in the label-to-BSX image translation. Furthermore, it is shown that the proposed method improves the performance regardless of the baseline network structure. Furthermore, comparison of the results of SPADE and pix2pix + LBP in Table A1 indicates that using a texture map following the proposed method is more efficient than increasing the model complexity. The parameters of the SPADE and pix2pix generators are 92.1 M and 41.8 M, respectively.

**Table A1:** FID according to the GAN models. (Resolution: 128 × 512).

Generated Image	FID	SWD
SPADE	100.4	1122.48
SPADE + LBP	59.3	1009.34
pix2pix	75.3	554.36
pix2pix + LBP	51.2	405.76
